# Avoiding bias in estimates of population size for translocation management

**DOI:** 10.1002/eap.2918

**Published:** 2023-09-28

**Authors:** Katherine T. Bickerton, John G. Ewen, Stefano Canessa, Nik C. Cole, Fay Frost, Rouben Mootoocurpen, Rachel McCrea

**Affiliations:** ^1^ Institute of Zoology, Zoological Society of London London UK; ^2^ School of Mathematics, Statistics and Actuarial Science University of Kent Canterbury UK; ^3^ Division of Conservation Biology, Institute of Ecology and Evolution University of Bern Bern Switzerland; ^4^ Durrell Wildlife Conservation Trust, Les Augrès Manor Jersey UK; ^5^ Mauritian Wildlife Foundation Vacoas Mauritius; ^6^ Department of Mathematics and Statistics Lancaster University Lancaster UK

**Keywords:** capture–recapture, conservation translocation, lesser night gecko, mark–recapture, *Nactus coindemirensis*, reintroduction

## Abstract

Mark–recapture surveys are commonly used to monitor translocated populations globally. Data gathered are then used to estimate demographic parameters, such as abundance and survival, using Jolly–Seber (JS) models. However, in translocated populations initial population size is known and failure to account for this may bias parameter estimates, which are important for informing conservation decisions during population establishment. Here, we provide methods to account for known initial population size in JS models by incorporating a separate component likelihood for translocated individuals, using a maximum‐likelihood estimation, with models that can be fitted using either R or MATLAB. We use simulated data and a case study of a threatened lizard species with low capture probability to demonstrate that unconstrained JS models may overestimate the size of translocated populations, especially in the early stages of post‐release monitoring. Our approach corrects this bias; we use our simulations to demonstrate that overestimates of population size between 78% and 130% can occur in the unconstrained JS models when the detection probability is below 0.3 compared to 1%–8.9% for our constrained model. Our case study did not show an overestimate; however accounting for the initial population size greatly reduced error in all parameter estimates and prevented boundary estimates. Adopting the corrected JS model for translocations will help managers to obtain more robust estimates of the population sizes of translocated animals, better informing future management including reinforcement decisions, and ultimately improving translocation success.

## INTRODUCTION

Conservation translocations are increasingly used in the conservation of threatened species (Seddon et al., 2014) and as part of ecological restoration programs (Ewen & Armstrong, 2008). Conservation translocation is defined as the deliberate movement of organisms from one site to another with beneficial outcomes at the population, species, or ecosystem level (IUCN/SSC, 2013). One of the key aims of a translocation, and a commonly used metric of success, is whether the survival of released individuals and their progeny allows the establishment and persistence of a new population (Armstrong & Seddon, 2008). Determining this requires intensive post‐release monitoring, this can be difficult when individuals are hard to detect (Sutherland et al., 2010). Low detection hampers the distinction between recruitment into a new population versus loss due to post‐release dispersal or mortality (Armstrong & Seddon, 2008; Converse et al., 2013). Additionally, translocated populations are initially small, meaning they can be at risk of Allee effects (Allee, 1931; Armstrong & Seddon, 2008).

Managers need estimates of population size and other vital rates to make decisions, especially during the early establishment phase, for example whether to stop or continue releases, or to provide additional in situ management (Armstrong & Seddon, 2008). To allow tracking and estimation, animals are usually marked to enable individual identification (e.g., colored bands, microchips, radiotransmitters, etc.) or photographed if individuals have unique markings. Individuals can then be surveyed using mark–recapture methodologies and demographic parameters estimated (Lebreton et al., 1992), such as survival and fecundity probabilities, and changes in abundance. One type of mark–recapture model is the Jolly–Seber (JS) model, which can be used to estimate probabilities of survival, capture, new entrants into the population and population size (Jolly, 1965; Seber, 1965). JS models are commonly used to monitor translocated populations (Aguirre et al., 2019; Dieterman et al., 2010; Dolny et al., 2018; Moseby et al., 2018). However, the small size of translocated populations, especially in the initial establishment phase, can create considerable uncertainty in abundance estimates (Hernandez et al., 2006). A further potential difficulty is that JS models assume that a proportion of the population is caught at each survey occasion. This assumption is violated initially in a translocated population as the first survey is the release, where all individuals in the population are recorded, and the capture probability is equal to 1. As far as we are aware, no examination has been made on how failure to account for the known number of individuals on the first occasion biases abundance estimates, nor has it been accounted for when modeling translocated populations.

In this study, we assess whether accounting for known initial population size affects abundance estimates in JS models. We tested this by constructing a modified JS model with separate likelihood components for translocated and wild‐born individuals that we compared using a standard JS model likelihood. We use a simulation study and a translocated population of lesser night gecko (*Nactus coindemirensis*) in Mauritius to compare the models. Our case study species was chosen due to its low capture probability, which can cause high levels of uncertainty in mark–recapture population estimates. Lesser night geckos are small, nocturnal, and cryptic, which makes them difficult to capture (Bullock et al., 1985; Cole et al., 2021). The lesser night gecko reintroduction is a typical example of a translocation within a species’ known historical range (IUCN/SSC, 2013) when long‐term mark–recapture studies have been undertaken post release. We provide methods to modify standard JS models in R (4.3, R Core Team, 2023) and MATLAB (MATLAB, 2023) to account for known initial population size. We show that not accounting for initial population size within JS models can lead to overestimates in abundance, particularly in species with a low detection probability.

## METHODS

### Translocation JS model

We propose a bespoke mark–recapture model that accounts for translocated individuals, building on the POPAN formulation of the JS model (Schwarz & Arnason, 1996), that we referred to as the translocation JS model. Let $h_{i}$ denote the encounter history of individual i which is born into the population and let $h_{i}^{*}$ denote the encounter history of a translocated individual i. Suppose individual i is first captured on occasion $f_{i}$ and last captured on occasion $\delta_{i}$. *x*_*ij*_ = 1 if individual i is captured at occasion j and $x_{\mathrm{ij}}=0$ otherwise. Let τ denote the occasion that new arrivals start entering the population, let D denote the number of observed individuals (including translocated individuals) and let $n_{0}$ denote the number of translocated individuals, and assume that these are ordered as the first $n_{0}$ of the D observed individuals.

We defined the parameters:

N: superpopulation of individuals born into the population (i.e., not including translocated individuals).

$\phi_{t}$: probability an individual who is alive at occasion *t* remains alive in the study area until occasion t+1 (often referred to as apparent survival probability).

$p_{t}$: probability an individual who is alive and in the study area at occasion t is captured on this occasion.

$\beta_{t-1}$: proportion of the superpopulation that arrives between occasions t−1 and t, and is first available for capture at occasion t. Note that $\beta_{j}=0$ for t=1,…,τ−1 and $\sum_{j=\tau }^{T}\beta_{j}=1$.

The likelihood for the joint model of translocated individuals and individuals hatched post translocation is defined by Equations ((1), (2), (3), (4)):

(1) $$L\left(N,\phi ,p,\beta ;x\right)\propto \frac{N!}{\left(N-D\right)!}\prod_{i=1}^{n_{0}}\mathrm{Pr}\left(h_{i}^{*}\right)\prod_{i=n_{0}+1}^{D}\mathrm{Pr}\left(h_{i}\right)\times \mathrm{Pr}{\left(h_{0}\right)}^{N-D},$$

where

(2) $$\mathrm{Pr}\left(h_{i}\right)=\sum_{b=\tau }^{f_{i}}\sum_{d=\delta_{i}}^{T}\beta_{b-1}\left(\prod_{j=b}^{d-1}\phi_{j}\right)\left(1-\phi_{d}\right)\left\{\prod_{j=b}^{d}{p_{j}}^{x_{\mathrm{ij}}}{\left(1-p_{j}\right)}^{1-x_{\mathrm{ij}}}\right\},$$

(3) $$\mathrm{Pr}\left(h_{i}^{*}\right)=\sum_{d=\delta_{i}}^{T}\left(\prod_{j=1}^{d-1}\phi_{j}\right)\left(1-\phi_{d}\right)\left\{\prod_{j=1}^{d}{p_{j}}^{x_{\mathrm{ij}}}{\left(1-p_{j}\right)}^{1-x_{\mathrm{ij}}}\right\},$$

(4) $$\mathrm{Pr}\left(h_{0}\right)=\sum_{b=1}^{T}\sum_{d=b}^{T}\beta_{b-1}\left(\prod_{j=b}^{d-1}\phi_{j}\right)\left(1-\phi_{d}\right)\left\{\prod_{j=b}^{d}\left(1-p_{j}\right)\right\}.$$

The likelihood function is then numerically optimized to evaluate the maximum‐likelihood estimates of parameters N, ϕ, p, and β. The derived estimates of $N_{t}$, the number of individuals in the population at occasion N, can be obtained by using the recursion (Equation 5):

(5) $$N_{t+1}=N_{t}{\hat{\phi }}_{t}+N{\hat{\beta }}_{t},$$

where $N_{1}=n_{0}$.

Confidence intervals for directly estimated and derived parameters are obtained using a nonparametric bootstrap procedure (DiCiccio & Efron, 1996). Group effects can be accommodated within this model, but it should be noted that separate estimates for N, denoted by $N_{g}$ will be obtained for each group and summed to give the total value of N given in the results (King & McCrea, 2019), because the likelihood (Equation 6) incorporating group effects will be:

(6) $$L\left(N,\phi ,p,\beta ;x\right)\propto \prod_{g=1}^{G}L\left(N_{g},\phi_{g},p_{g},\beta_{g};x_{g}\right),$$

where $x_{g}$ denotes the encounter histories for individuals from group g and the parameters with subscript g denote the parameters as defined above but with group dependence.

The translocation JS model can be fitted by constraining the POPAN JS model, such that the initial values of p and β are set to 1 and 0, respectively, until it is biologically feasible for new entrants to have joined the population. In the following analyses, we fitted these models using our bespoke code and a standard unconstrained POPAN formulation of the JS model. We also compared estimates from these models with those from mark–recapture R packages *rMark* (Laake, 2013) and *marked* (Laake et al., 2013). Parameters can be generalized to include temporal ($\gamma_{t}$) covariates, such that$\;\text{logit}\left(\phi_{\mathrm{it}}\right)=\theta_{1}+\theta_{2}\gamma_{t}$.

### Simulation study

To compare our translocation JS model with the unconstrained POPAN formulation of the JS model (hereafter referred to as the standard JS model), we simulated capture histories of translocated populations based on a standard range of parameter values from the literature. The parameters N, β, ϕ, and p can all be estimated from mark–recapture survey data using the POPAN formulation of the JS model (Schwarz & Arnason, 1996). By simulating populations, we were able to compare known parameter values with model estimates and assess the accuracy of estimates in different scenarios.

We tested 12 translocation scenarios (Table 1) to reflect the variety of situations commonly encountered in conservation translocations. All simulated data were in the form of capture histories across 10 evenly spaced 6‐month intervals. We varied $N_{1}$ between 15 and 30 and N between 500 and 2000. For all scenarios, we set monthly ϕ to be broadly applicable to all vertebrate species using a random uniform distribution between 0.94 and 0.99 (bounds are upper and lower quartiles of vertebrates in the DatLife database; DatLife, 2021) to allow for stochasticity between surveys. Because the translocation release is counted as the first survey occasion, the initial p=1, as all individuals in the population are detected during this “survey.” The remaining values of p were simulated using a random uniform distribution within intervals specific to the scenario (low = 0.1–0.3; mid = 0.4–0.6; high = 0.7–0.9) as p is highly variable across different animal species. A range was again used to allow for stochasticity. Entry probabilities must sum to 1 within JS models and the first entry β_0_ is the proportion of the total population translocated ($\frac{N_{1}}{N}$). In most translocations, it will be several surveys before new entrants are recorded in the adult population, therefore we set $\beta_{1,2}=0$, the remaining values were generated from a random uniform distribution between 0.1 and 0.2, then scaled to ensure the values summed to 1 for the survey occasions $\beta_{3:K}$. This delay is dependent on the life history of the species being translocated (i.e., gestation period, life stages, recruitment time), the time between surveys and whether nonadult life stages are included as part of the population being estimated.

**TABLE 1:** Parameter values used for each simulation scenario: total population size (*N*), number of individuals translocated therefore initial population size (*N*_1_), entry probability (β), annual survival probability (ɸ), and capture probability (*p*).

Scenario	*N*_1_	*N*	β ^a^	ɸ^a^	*p* ^a^
1	15	500	0.1–0.2	0.94–0.99	0.1–0.3
2	15	500	0.1–0.2	0.94–0.99	0.4–0.6
3	15	500	0.1–0.2	0.94–0.99	0.7–0.9
4	30	500	0.1–0.2	0.94–0.99	0.1–0.3
5	30	500	0.1–0.2	0.94–0.99	0.4–0.6
6	30	500	0.1–0.2	0.94–0.99	0.7–0.9
7	15	2000	0.1–0.2	0.94–0.99	0.1–0.3
8	15	2000	0.1–0.2	0.94–0.99	0.4–0.6
9	15	2000	0.1–0.2	0.94–0.99	0.7–0.9
10	30	2000	0.1–0.2	0.94–0.99	0.1–0.3
11	30	2000	0.1–0.2	0.94–0.99	0.4–0.6
12	30	2000	0.1–0.2	0.94–0.99	0.7–0.9

^a^ Where ranges of values are given, values were selected randomly from a uniform distribution in the interval of these values.

To simulate capture histories, we first simulated presence histories by randomly assigning when each individual entered the population using a random multinomial distribution with a probability of $\beta_{k}$ (k = survey occasion). We then determined whether the individual survived to the next survey occasion using a Bernoulli distribution with a probability of $\phi_{k}$. If the individual did not survive, it was removed and the remainder of the surveys were marked as 0 for absence. From each individual's presence history, we then simulated a capture history. For each occasion in which the individual was present, we used a Bernoulli distribution with a probability of $p_{k}$ to determine whether the individual was caught and marked as 1 for seen, and 0 for not seen.

We simulated each scenario 250 times, and estimated values of N, β, ϕ, and p using the translocation and standard JS models, with constant N, ϕ, and p and time dependent β (from which $N_{t}$ was calculated by recursion). We calculated the difference between the simulated parameter values and the estimates from the translocation and standard JS models. Both models can be run using R (R Core Team, 2023) or MATLAB (MATLAB, 2023); we performed our simulations in MATLAB because it allowed faster optimization.

### Case study

**FIGURE 1:**
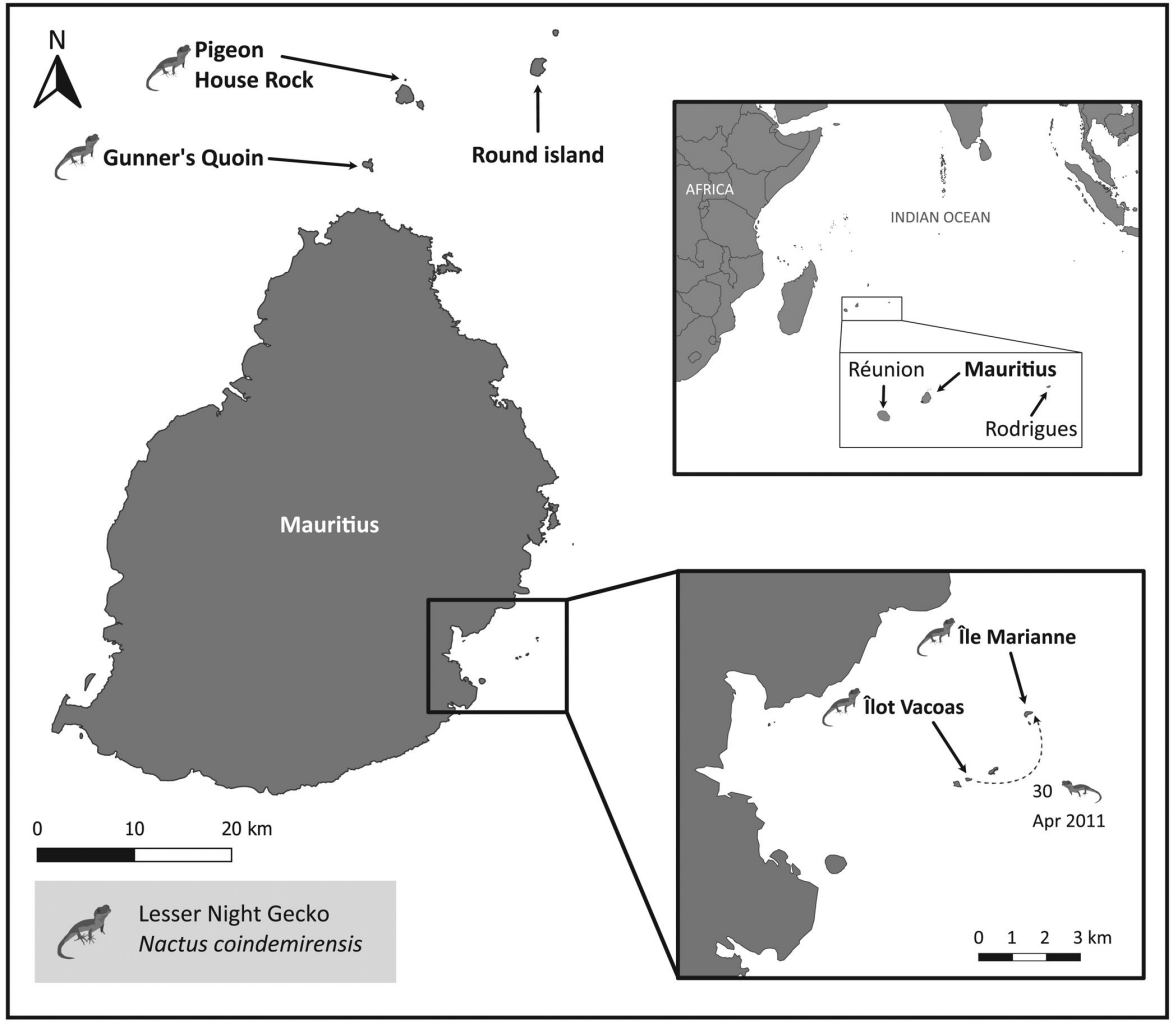
Map of Mauritius and outlying Mauritian islands indicating translocations between islands. The dotted line indicates the translocation of lesser night geckos (*Nactus coindemirensis*) with the number of individuals translocated and the month and year of translocation.

To demonstrate the implementation of the translocation JS model for a real‐world case study, we fitted the model to a dataset of the lesser night gecko, one of three endemic *Nactus* species found in Mauritius (Arnold, 2000; Arnold & Jones, 1994) and currently classified as “Vulnerable” by the IUCN Red List (Cole et al., 2021). Lesser night geckos are nocturnal, elusive, and the smallest of the Mascarene *Nactus* species with adult snout‐to‐vent length (SVL) of 30.9 mm ± 1.4 (SE) in males and 33.7 mm ± 1.6 in females (Goble & Goetz, 2011). Individuals are uniquely identifiable by their dark brown dorsal pattern, making the species a suitable candidate for mark–recapture surveys (Figure 1). Lesser night geckos were likely to be widespread across Mauritius prior to European colonization in the 16th century (Arnold, 1980; Cole et al., 2005). However, human colonization, resulting in habitat destruction and the introduction of nonnative mammal and reptile species, caused the decline and loss of lesser night gecko populations from most of their range (Cole et al., 2005). They are now restricted to three offshore islands: Gunner's Quoin (72.9 ha), Ilot Vacoas (1.1 ha), and Pigeon House Rock (1.4 ha) (Cole et al., 2021; Appendix S1: Table S1), as well as a captive population at Durrell Zoo, Jersey, Channel Islands, UK (Figure 1).

A translocation of 75 lesser night geckos was carried out in April 2011 to Ile Marianne, a 2.1‐ha island 6.15 km off the SE coast of Mauritius. Wild individuals from nearby Ilot Vacoas (2.4 km southwest of Ile Marianne) were caught and processed (photographed, weighed, measured, and sexed) on 13 April, then translocated by boat and released upon arrival the same night ($n=30,n_{\text{male}}=15,n_{\text{female}}=15$). Captive‐bred individuals from Durrell Zoo, Jersey, and 45 eggs were transported via plane from Jersey to Mauritius, arriving on 14 April. Individuals were checked before departure and upon arrival by respective government vets. They were held in a biosecure facility until the evening of arrival, processed as above, then transported via boat to Ile Marianne and released immediately ($n=45,n_{\text{male}}=11,n_{\text{female}}=20,n_{\text{juvenile}}=14$). Eggs were placed in predator‐proof nest boxes within suitable habitats at the same time as the captive‐bred geckos were released.

To monitor population dynamics, 19 mark–recapture surveys have been carried out to date, at least annually since April 2011 (except in 2020, when no surveys could be carried out due to the COVID‐19 pandemic). Each survey ran for 2–4 nights. Each night, the same route was walked through the areas of suitable habitats and all lesser night geckos found were caught, processed, and checked for injuries. Recaptures across the multiple nights within a survey were combined such that each individual was either seen (1) or not (0) on each survey occasion. Ideally, surveys were carried out on dry nights, at least 1 h after the sun had set fully and where possible avoiding a full moon, to maximize capture probability as the species is most active in these conditions. Surveying in ideal conditions is not always possible, therefore temperature and moon phase were recorded at the start of each night. Moon phase and temperature were averaged for each survey (temperatures = mean, moon phase = mode). The same two observers were involved in all 19 surveys. Individual recapture histories were generated by comparing the unique dorsal patterns (between front and hind legs; Appendix S1: Figure S1) using the photo ID software “Hotspotter” (Crall et al., 2013). Only adults were considered in our recapture histories due to a low detection probability and high risk of injury during the capture of juveniles (no. unique adults = 475). Entrants to the adult population were either those that hatched on the island or juveniles that were released during the translocation that are now of adult size (recruitment occurs at ~6 months).

We used the translocation and standard JS models to estimate N, β, ϕ, and p for the lesser night gecko population. We also used the POPAN model in the R package rMark and the JS model in the R package marked. For all models, $N_{t}$ was calculated from the estimated parameters. Uneven time intervals between surveys were accounted for within the likelihood for ϕ (in months) and by allowing β to be fully time dependent. The release was included as the first survey occasion. We assessed the variation in all parameters against time, using a monthly scale as the lifespan of the species is 3–4 years (Cole et al., 2021; Goble & Goetz, 2011). We also examined whether β, ϕ, and p were constant or varied with sex, where individual covariates were modeled as groups. Finally, we looked for the variation in p with air temperature, survey effort, substrate temperature and moon phase. Air and substrate temperature are correlated, therefore air temperature only was used as more data became available. Survey effort was defined as the number of survey periods for each trip. All covariates were modeled using a log link for N, a multinomial logit link for β and a logit link for ϕ and p. Although each survey consisted of multiple days, a robust design model was not appropriate in this case, as there were very few recaptures of individuals within each survey.

We fitted models and completed model selection by optimizing the likelihoods of the translocation and standard JS models with all possible combinations of covariates. As model outputs should be the same for the standard JS model and the R packages used, running the selection process in all would have been redundant. Top models were selected using Akaike's Information Criterion (AIC). A comparison of software was carried out using a standard JS model, our translocation JS model, the POPAN model in the R package *rMark* and the JS model in the R package *marked*. The same model was run in each (β~time,ϕ~time,p~1) and bootstrap confidence intervals were used.

## RESULTS

### Simulation results

**FIGURE 2:**
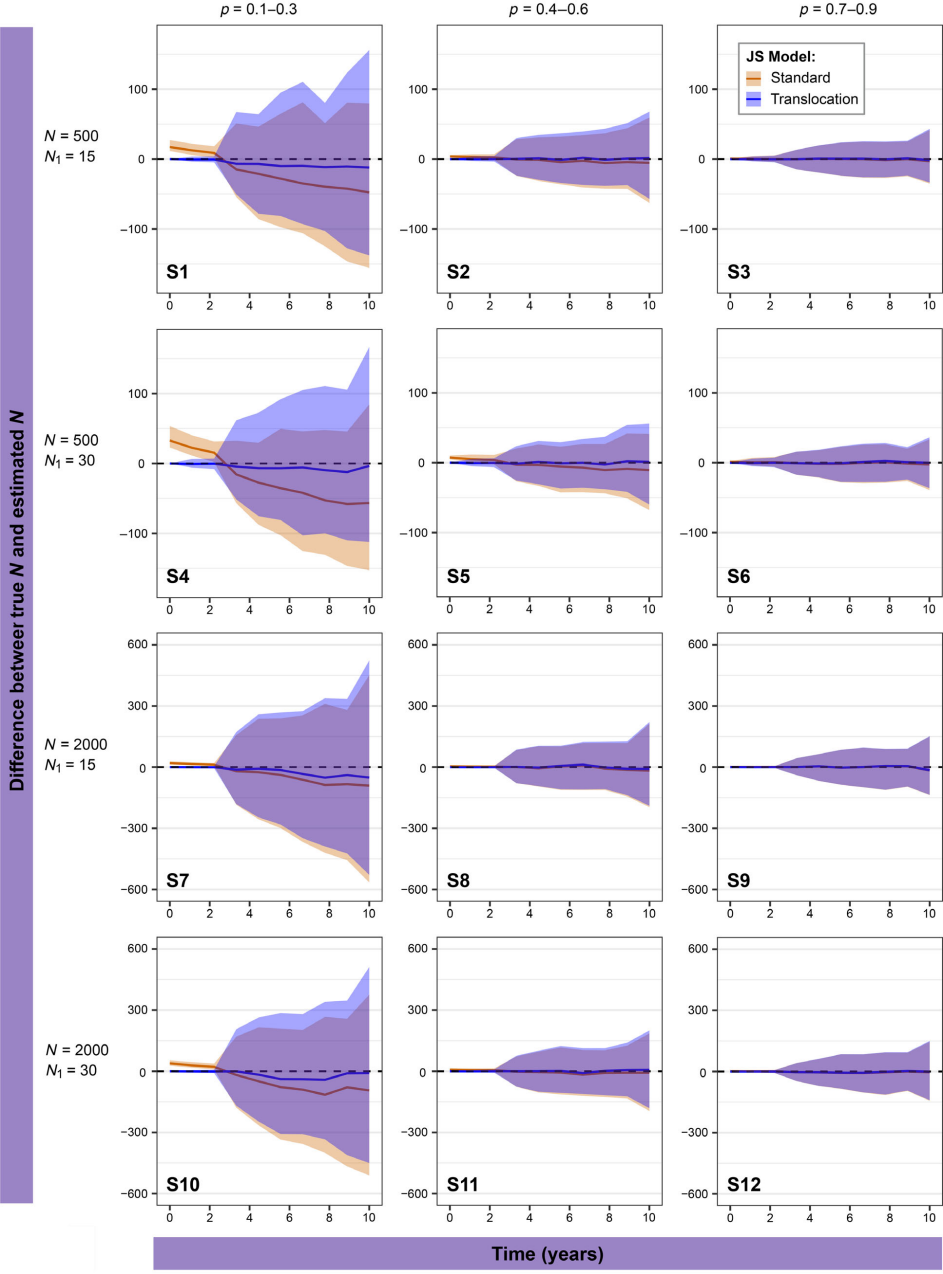
Differences between the estimates of population size *N*_*t*_ by two models and the true simulated value across 12 scenarios (Table 1). Modeled with entry probability as a function of time and survival and capture probabilities as constant. Standard Jolly–Seber (JS) model in orange and translocation JS model in blue with 95% CIs.

The standard JS model substantially overestimated $N_{t}$ in the first 2 years when capture probability (p) was below 0.3 whereas our translocation JS model avoided this by giving estimates much closer to the true value (Figure 2: S1, S4, S7, and S10). Both JS models were fitted using time‐dependent arrival probability (β) and constant population size (N), survival probability (ϕ) and p. In the first 2 years, the standard JS model had a percentage difference of 78%–130% for the low p scenarios, as opposed to a 0%–8.9% difference in the translocation JS model. After the first 2 years, estimates were more accurate with percentage differences of 5.2%–23% and 8.9%–0.1% for standard and translocation models, respectively (Appendix S2: Table S1). This was due to p being constant where initially we know it should be 1, therefore the averaging of values across surveys led to an initial underestimation, then overestimation in survey occasions following the release. This can be overcome by making parameters fully time dependent; however, this increases the likelihood of boundary estimates and parameter redundancy, especially with small sample sizes as we observed in our preliminary simulations and in our case study.

The accuracy of $N_{t}$ estimates increased with increasing capture probabilities. In scenarios where p = 0.1–0.3, the average percentage differences between true values and estimates were 22.2% and 4.1% for standard JS and translocation JS models, respectively. In our medium, detection scenarios, where p = 0.4–0.6, initial estimates of $N_{t}$ were slightly inflated and confidence intervals were larger in the standard JS model compared with our translocation JS model (Figure 2: S2, S5, S8 and S11) but not on the same scale as lower p values, as the percentage differences between true and estimated values were 6% and 0.2% for standard and translocation JS models, respectively. Estimates of $N_{t}$ were most accurate in scenarios with high p (0.7–0.9) where the difference between the standard and translocation JS model were negligible with both models having a percentage difference of 0.5% between true and estimated values (Figure 2: S3, S6, S9, and S12; Appendix S2: Table S1).

A larger initial population size increased the accuracy of $N_{t}$. Simulation scenarios with an initial population size of 15 (Figure 2: S1–S3 and S7–S9) had higher average percentage differences in both models (standard JS = 10.4%, translocation JS = 1.7%) compared with scenarios with an initial population size of 30 (standard JS = 8.8%, translocation JS = 1.4%; Figure 2: S4–S6 and S10–S12). Accuracy did not differ between superpopulation sizes.

**FIGURE 3:**
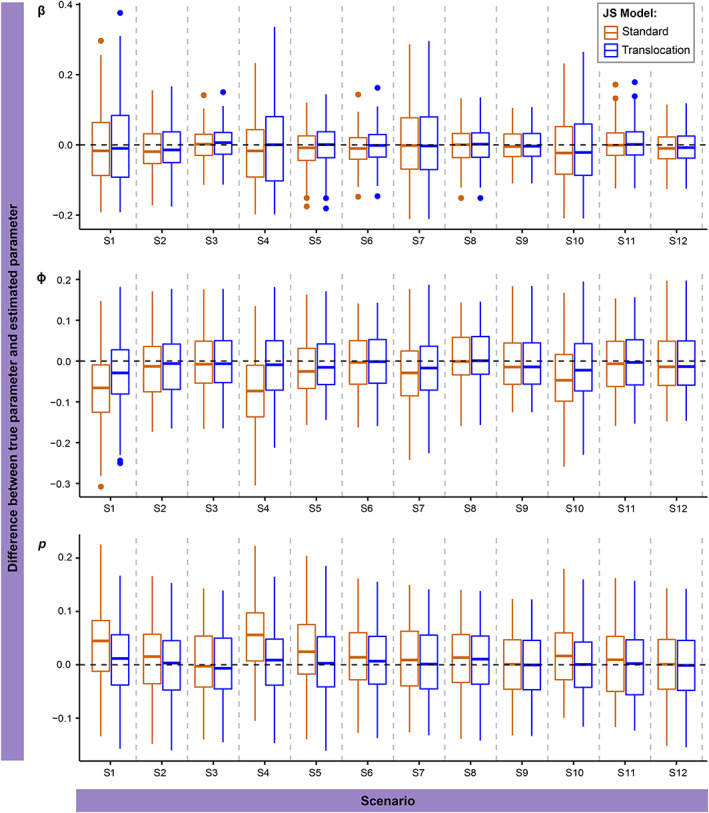
Difference between estimates and true simulated values of entry probability β, survival probability ɸ and capture probability *p* from the standard Jolly–Seber (JS) model (orange) and the translocation JS model (blue) across 12 scenarios (Table 1). Modeled with entry probability as a function of time and survival and capture probabilities as constant. Boxplots show median, upper and lower quartiles, and range.

Estimates of β did not differ significantly between standard and translocation JS models (Figure 3). In the simulated data, β was fixed to 0 for times 2 and 3, to replicate the delay in new entrants to the population post translocation, which was accounted for in our translocation JS model. In the standard JS model, when β was modeled as time dependent, estimates for times 2 and 3 tended toward 0. As with $N_{t}$, the uncertainty of estimates was lowest in scenarios with the highest p. There was no significant difference in β estimates between high and low superpopulation or initial population scenarios.

In low p scenarios, ϕ was underestimated (Figure 3: S1, S4, S7, and S10) by both models. The translocation JS model estimates were more accurate in all scenarios, although this difference was small in medium and high p scenarios (Figure 3). In the low p scenarios, the percentage difference of the translocation JS models from the true value was between 1.4% and 3.6% compared with 4.2%–8.5% for the standard JS model. Estimates of ϕ from the standard JS model were less accurate in scenarios with smaller superpopulation sizes but did not vary between initial population sizes. The translocation JS model ϕ estimates had a similar accuracy with differing superpopulation and initial population size.

Detection probability p was overestimated by both models in low p scenarios (Figure 3: S1, S4, S7, and S10) but only the standard JS model in medium p scenarios (Figure 3: S2, S5, S8 and S11) and overestimations were higher in scenarios with smaller superpopulation size. For low p and low superpopulation scenarios, percentage difference between true and estimated values was between 9.8% and 16% for the standard JS and 3.3%–5.1% for the translocation JS model, compared with 0.9%–1.2% (standard) and 1.6%–3.1% (translocation) for the low p and high superpopulation models. Initial population size did not affect estimates of p.

### Case study results

**FIGURE 4:**
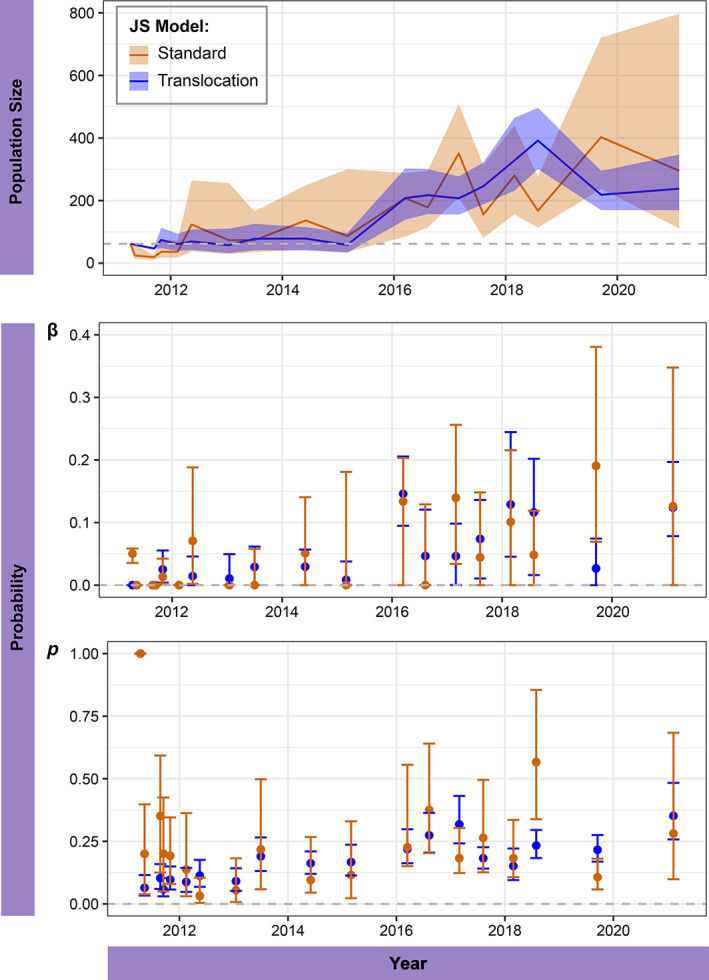
Comparison of parameter estimates for population size *N*_*t*_, entry probability β and capture probability *p* from top fitting models (lowest AIC value) for the standard Jolly–Seber (JS) model (orange) and the translocation JS model (blue) for the lesser night gecko (*Nactus coindemirensis*) population on Ile Marianne, Mauritius. Estimates from translocations in April 2011 until most recent survey in June 2021. Initial population size (*n* = 62) is indicated in top panel (gray dashed line). Both top models had sex dependent superpopulation *N* and time dependent β, the standard JS model had time dependent ɸ and *p*, the translocation JS model had constant ɸ and *p* dependent on survey effort, air temperature, and moon phase.

The model with the lowest value of AIC, and thus the most favored model for our translocation JS formulation, had time dependent β, constant ϕ, and capture that varies with survey effort, moon phase, and air temperature (see Appendix S3: Table S1 for full model selection). The lowest AIC for our standard JS formulation resulted from β, ϕ, and p all being time dependent (see Appendix S3: Table S2 for full model selection). In the period prior to new entrants joining the population (β=0), estimates of $N_{t}$ were lower for the standard JS compared with the translocation JS (Figure 4). The lack of the overestimates that were observed in our simulation study was due to the time dependence in β, ϕ, and p, preventing the overestimation of the initial population size. Once new entrants started joining the population, the standard JS model estimated a sudden increase and a highly variable population size. The translocation JS model estimated a similar average population size to the standard JS model with a more gradual increase and lower uncertainty.

Estimates of β, ϕ, and p from the standard and translocation JS models were similar, however the translocation JS model consistently has lower uncertainty and more gradual variations in estimated values than the standard JS model (Figure 4). Estimated values of β were similar for both models and despite only the translocation model accounting for the period of no new entrants initially, both models estimated initial β=0 then increased with time. Estimates of ϕ followed a similar trend in both models with high survival of translocated individuals initially, then variation across time. The standard JS model, which was fully time dependent, gives boundary estimates (ϕ=1) for seven of the survey periods, and confidence intervals are consistently very broad. This does not occur in the translocation JS model where annual ϕ was constant (ϕ=0.514[0.452−0.575]). In the translocation JS model, p was fixed to 1 initially to account for the translocation, the standard JS model, although not fixed, also estimated the value of $p_{1}=1$. The translocation JS model estimated that p increased linearly with survey effort and air temperature and was highest when the moon phase was between crescent and gibbous, and lowest close to new and full moons.

A comparison of models was carried out using a standard JS model, our translocation JS model (both run using MATLAB), the POPAN model in the R package *rMark* and the JS model in the R package *marked* (Appendix S3: Figures S1–S3). We encountered optimization issues with both R packages with and without fixed parameters, especially when attempting to fix the initial capture probability to 1, leading to boundary estimates. Abundance estimated from *rMark* and *marked* both dropped below one individual in the first year post release (Appendix S3: Figure S1). The parameter estimates and confidence intervals from *rMark* were very similar to the standard JS model and translocation JS model, with similar final estimates of abundance, and greater variance in estimates of β and ϕ although following the same pattern (Appendix S3: Figures S2 and S3). The final abundance estimate from *marked* was much lower in comparison with broader confidence intervals (Appendix S3: Figure S1) and there were more boundary estimates of β and ϕ (Appendix S3: Figures S2 and S3) indicating issues with optimization.

## DISCUSSION

Our results demonstrated that the standard POPAN formulation of the JS model is likely to overestimate population size when fitted to translocated populations, especially in challenging translocations with small numbers of founders and low detection probabilities. Unfortunately, low detection and few founders are common features of many conservation translocations, which then frequently need ongoing management to support population establishment. To our knowledge, although JS models are commonly used to estimate the abundance of translocated populations, accounting for known initial population size in JS models for translocated populations is not standard practice. However, overestimating population size can have serious implications for conservation management decisions that rely on accurate estimates. In both our simulations and case study, accounting for initial population size within JS models through a translocation‐specific likelihood substantially improved estimates. Our case study demonstrated that separating the translocated individuals can allow covariates to explain the trends in the data as opposed to purely time dependence, reducing the number of parameters and therefore the likelihood of parameter redundancy.

In the case of the lesser night gecko (*Nactus coindemirensis*), a cryptic threatened species with low detectability, we were able to avoid overestimation even with the standard JS model, by fitting a fully time‐dependent model (i.e., one where β, ϕ and p varied by time). However, a fully time dependent formulation has a high number of parameters (k=60) and so is more likely to encounter parameter redundancy, as we saw with the boundary estimates in survival (Figure 4). More parameters increase the chance of estimates with high uncertainty and low accuracy. In contrast, when we fitted our translocation JS model, the AIC supported simpler models, and the top‐ranked model had fewer parameters (k=22). This was achieved through being able to use covariates to explain patterns in capture, allowing optimization of future surveys (Broder et al., 2020). The reduced uncertainty and lack of boundary estimates produce realistic estimates of population size from this model.

On the one hand, overestimated population size can generate unfounded optimism in reintroduction success. In such cases, if managers believe the population is larger than it really is, they might miss a crucial window of opportunity to reinforce it, thereby averting possible establishment failure (Armstrong & Seddon, 2008; Panfylova et al., 2019) driven by stochastic demographic processes (Bubac et al., 2019; Clark et al., 2002; Converse et al., 2013; Griffith et al., 1989), dispersal (Moseby et al., 2014; Resende et al., 2021) or Allee effects (Allee, 1931; Armstrong & Wittmer, 2011; Courchamp et al., 1999). Even if the population persists without reinforcement, it may be at increased risk of longer term inbreeding depression and reduced adaptive potential driven by this and genetic drift (Balestrieri et al., 2021; Frankel, 1970; Frankham, 2005).

On the other hand, underestimating population size might lead to the program either prematurely giving up on supporting the establishing population or investing in added but unnecessary management (Armstrong & Wittmer, 2011). For example, if managers mistakenly believe a population to be at risk, they might undertake reinforcements that impact source populations (Dimond & Armstrong, 2007; Earnhardt et al., 2014; Turko et al., 2021) and incur additional resource commitments (Berger‐Tal et al., 2020; Bubac et al., 2019; Crimmins et al., 2009). Individuals released in reinforcement translocations may face unexpected resistance to recruitment into the reintroduced population due to territorial aggression from residents from earlier releases.

Accurate estimates of population size and the parameters from which they are derived are therefore important for good state‐dependent management decisions, as the growth or decline of populations are key metrics in guiding future management actions (Armstrong & Seddon, 2008; Seddon et al., 2007). Additionally, if planned in accordance with the IUCN guidelines, most translocations are likely to have specific demographic targets or trigger points at which specific management actions are taken (Armstrong & Ewen, 2001; Chades et al., 2008). Minimizing uncertainty is important to ensure management actions are timely (Converse et al., 2013; Panfylova et al., 2019).

We note that a simple constraint to the initial population size could be easily implemented in a Bayesian framework. However, our model goes beyond simply constraining the model, instead allowing a separate likelihood component for translocated individuals that can be used for single or multiple translocation events, for example in the event of subsequent reinforcements. These likelihood components do not directly contribute to the estimation of population size as the model is derived by conditioning on these known releases. Moreover, Bayesian inference is still not necessarily accessible to all ecologists, and much management inference relies on maximum‐likelihood‐based software such as the program *MARK* or the related R packages we considered in our analysis.

When estimating the abundance of translocated populations with low capture probability, especially in the early stages of the translocation, uncertainty can be high and models less informative, which can result in poor decisions. In such situations, we recommend accounting for known initial population size by use of our translocation JS model, to increase the accuracy of parameter estimates, better inform future management decisions, and increase the chance of successful establishment and persistence.

## CONFLICT OF INTEREST STATEMENT

The authors declare no conflicts of interest.

## Supporting information


Appendix S1.

Appendix S2.

Appendix S3.

## Data Availability

Data and code (Bickerton et al., 2023) are available in Zenodo at https://doi.org/10.5281/zenodo.8215314.
