# Supplementary material for: Avoiding bias in estimates of population size for translocation management
Source: Ecol Appl. 2023 Sep 28;33(8):e2918. doi: 10.1002/eap.2918 (PMC10909443; doi:10.1002/eap.2918)
Supplement: Supplementary file 1 — Appendix S1. [file EAP-33-e2918-s003.pdf]

Appendix S1: Case Study extra information

Authors: Katherine T. Bickerton, John G. Ewen, Stefano Canessa, Nik C. Cole, Fay Frost, Rouben Mootoocurpen, Rachel McCrea

Manuscript title: Avoiding bias in estimates of population size for translocation management.

Journal name: Ecological Applications

Table S1: Geographical information about the study system islands.

Island	Area (ha)	Area (km^2)	Latitude	Longitude
Gunner's Quoin	72.9	0.73	-19.94133	57.61998
Île Marianne	2.1	0.02	-20.38019	57.78723
Ilot Vacoas	1.1	0.01	-20.39776	57.77053
Pigeon House Rock (Rocher aux Pigeons)	1.4	0.01	-19.86271	57.65740

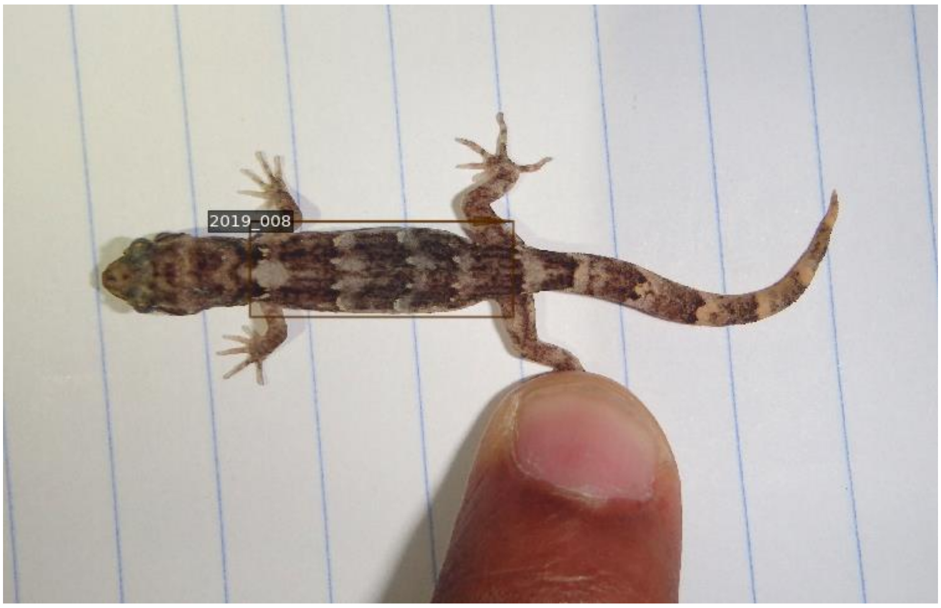

Figure S1: Example photo from mark recapture surveys of lesser night gecko (*Nactus coindemirensis*). The region used in photo identification is indicated by the brown rectangle and unique individuals are identified using *Hotspotter* (Crall *et al.* 2013), which allocated a unique reference code to each individual, as shown. Photo credit Nik Cole.

References

Crall, J. P., Stewart, C. V., Berger-Wolf, T. Y., Rubenstein, D. I. and Sundaresan, S. R. 2013. *HotSpotter — Patterned species instance recognition.* 2013 IEEE Workshop on Applications of Computer Vision (WACV): 230-237. DOI: 10.1109/WACV.2013.6475023.
