# Supplementary material for: Avoiding bias in estimates of population size for translocation management
Source: Ecol Appl. 2023 Sep 28;33(8):e2918. doi: 10.1002/eap.2918 (PMC10909443; doi:10.1002/eap.2918)
Supplement: Supplementary file 2 — Appendix S2. [file EAP-33-e2918-s002.pdf]

Appendix S2: Simulation Results

Authors: Katherine T. Bickerton, John G. Ewen, Stefano Canessa, Nik C. Cole, Fay Frost, Rouben Mootoocurpen, Rachel McCrea

Manuscript title: Avoiding bias in estimates of population size for translocation management.
Journal name: Ecological Applications

Table S1: Simulated and estimated average values of population size over time *Nt*. Simulated values are labelled as true, estimates from the standard Jolly-Seber model as JS and estimates from the translocation Jolly-Seber model as T. Percentage differences were calculated between simulated and estimated values for standard and translocation Jolly-Seber models, indicated as *% Diff*.

Scenario	Time	True Nt	Nt T	Nt JS	% Diff T	% Diff JS
**S1**	1	15	15	32	0.000	1.150
	2	13	12	25	-0.080	0.902
	3	10	10	19	-0.046	0.877
	4	80	73	65	-0.079	-0.180
	5	135	130	116	-0.037	-0.139
	6	181	168	149	-0.073	-0.175
	7	221	203	181	-0.077	-0.178
	8	248	236	211	-0.045	-0.146
	9	267	252	223	-0.057	-0.164
	10	290	276	243	-0.048	-0.160
**S2**	1	15	15	19	0.000	0.253
	2	12	12	15	0.028	0.278
	3	10	10	13	0.015	0.251
	4	79	79	78	-0.004	-0.013
	5	137	138	135	0.004	-0.013
	6	181	180	178	-0.006	-0.018
	7	217	216	212	-0.002	-0.021
	8	247	247	242	0.000	-0.020
	9	268	273	267	0.018	-0.004
	10	290	291	284	0.003	-0.021
**S3**	1	15	15	16	0.000	0.047

	2	13	12	13	-0.051	-0.009
	3	11	10	11	-0.078	-0.038
	4	77	76	76	-0.007	-0.008
	5	132	131	131	-0.006	-0.008
	6	179	181	180	0.013	0.010
	7	215	212	212	-0.010	-0.013
	8	246	245	244	-0.003	-0.007
	9	271	271	270	0.004	-0.002
	10	294	292	290	-0.007	-0.013
**S4**	1	30	30	63	0.000	1.097
	2	25	24	47	-0.025	0.890
	3	20	20	36	-0.010	0.780
	4	85	77	67	-0.089	-0.216
	5	137	129	105	-0.055	-0.231
	6	181	174	145	-0.038	-0.199
	7	215	208	170	-0.035	-0.212
	8	241	234	197	-0.027	-0.183
	9	266	255	211	-0.041	-0.208
	10	282	281	231	-0.002	-0.181
**S5**	1	30	30	37	0.000	0.247
	2	25	25	30	-0.014	0.216
	3	21	20	25	-0.012	0.199
	4	85	83	81	-0.028	-0.047
	5	134	134	130	0.000	-0.026
	6	177	176	171	-0.005	-0.035
	7	213	213	207	0.000	-0.029
	8	245	244	236	-0.003	-0.036
	9	269	271	262	0.007	-0.027
	10	291	287	276	-0.011	-0.052
**S6**	1	30	30	31	0.000	0.044
	2	25	25	26	-0.016	0.027
	3	20	20	21	0.009	0.050
	4	82	79	79	-0.031	-0.032
	5	136	133	133	-0.015	-0.018
	6	177	175	174	-0.011	-0.016
	7	212	214	212	0.007	0.001
	8	239	241	239	0.008	0.001
	9	265	267	265	0.010	0.001
	10	286	286	284	0.003	-0.007
**S7**	1	15	15	35	0.000	1.304
	2	13	12	28	-0.069	1.117

	3	10	10	22	-0.023	1.187
	4	287	271	261	-0.056	-0.091
	5	517	495	476	-0.042	-0.079
	6	705	695	668	-0.014	-0.052
	7	870	836	806	-0.039	-0.073
	8	1007	957	924	-0.049	-0.083
	9	1120	1076	1033	-0.039	-0.078
	10	1209	1131	1088	-0.064	-0.100
**S8**	1	15	15	19	0.000	0.261
	2	13	12	15	-0.056	0.192
	3	10	10	13	0.005	0.265
	4	297	293	292	-0.011	-0.015
	5	520	523	521	0.007	0.003
	6	700	702	699	0.003	0.000
	7	864	872	867	0.010	0.003
	8	997	997	991	0.000	-0.006
	9	1114	1094	1087	-0.018	-0.024
	10	1192	1181	1174	-0.009	-0.015
**S9**	1	15	15	16	0.000	0.047
	2	12	12	13	0.031	0.079
	3	10	10	11	0.021	0.066
	4	299	289	289	-0.032	-0.032
	5	521	529	529	0.017	0.016
	6	717	715	715	-0.002	-0.003
	7	875	877	876	0.002	0.002
	8	1001	1000	999	-0.001	-0.002
	9	1119	1112	1110	-0.007	-0.008
	10	1208	1196	1194	-0.010	-0.011
**S10**	1	30	30	69	0.000	1.295
	2	25	24	53	-0.032	1.132
	3	21	20	42	-0.070	0.994
	4	303	292	276	-0.037	-0.089
	5	528	514	485	-0.027	-0.082
	6	716	675	632	-0.058	-0.118
	7	868	811	757	-0.065	-0.127
	8	988	935	876	-0.054	-0.113
	9	1085	1065	1003	-0.018	-0.075
	10	1175	1145	1065	-0.025	-0.093
**S11**	1	30	30	38	0.000	0.264
	2	25	25	31	-0.010	0.242
	3	20	20	25	0.021	0.273
	4	290	280	278	-0.033	-0.039

	5	511	507	502	-0.007	-0.016
	6	702	700	694	-0.002	-0.011
	7	858	856	847	-0.002	-0.012
	8	991	994	983	0.004	-0.008
	9	1104	1104	1092	0.000	-0.011
	10	1174	1196	1181	0.019	0.006
**S12**	1	30	30	31	0.000	0.047
	2	25	25	26	-0.019	0.029
	3	21	20	21	-0.022	0.023
	4	289	282	282	-0.023	-0.023
	5	526	529	529	0.007	0.005
	6	715	709	708	-0.009	-0.010
	7	878	867	866	-0.012	-0.013
	8	997	1003	1002	0.007	0.005
	9	1097	1094	1092	-0.003	-0.004
	10	1171	1173	1171	0.002	0.000
