# Supplementary material for: Avoiding bias in estimates of population size for translocation management
Source: Ecol Appl. 2023 Sep 28;33(8):e2918. doi: 10.1002/eap.2918 (PMC10909443; doi:10.1002/eap.2918)
Supplement: Supplementary file 3 — Appendix S3. [file EAP-33-e2918-s001.pdf]

Appendix S3: Case Study Additional Results

Authors: Katherine T. Bickerton, John G. Ewen, Stefano Canessa, Nik C. Cole, Fay Frost, Rouben Mootoocurpen, Rachel McCrea

Manuscript title: Avoiding bias in estimates of population size for translocation management.

Journal name: Ecological Applications

Table S1: Model selection table for translocation likelihood model, indicating formulas for each parameter: population size *N*, entry probability β, survival probability ϕ and capture probability *p*. Log likelihood *LogL*, number of parameters *k*, Akaike's Information Criterion *AIC*, and difference between top ranked model (by AIC) and all other models *ΔAIC*, is given for each model. The top 10 best fitting models (by AIC) are given as well as the models where either ϕ or *p* are constant or time dependent (where these were not within the 10 best models).

N	$\boldsymbol{\beta}$	$\boldsymbol{\phi}$	***p***	***LogL***	***k***	***AICc***	***ΔAICc***
Sex	Time	1	Temp + Moon + Effort	-422.85	22	-801.69	0
Sex	Time	Sex	Temp + Moon + Effort	-423.20	23	-800.39	1.30
Sex	Time	1	Sex + Temp + Moon + Effort	-425.54	26	-799.08	2.61
Sex	Time	Time	Temp + Effort	-437.19	39	-796.37	5.32
Sex	Time	Time	Effort	-436.07	38	-796.14	5.55
Sex	Time	Time	Temp	-435.75	38	-795.49	6.20
Sex	Time	Time	1	-434.38	37	-794.76	6.93
Sex	Time	Time	Temp + Moon + Effort	-437.26	40	-794.53	7.16
Sex	Time	Time	Moon + Effort	-436.07	39	-794.14	7.55
Sex	Time	Time	Temp + Moon	-435.76	39	-793.53	8.16
Sex	Time	Time	Time	-451.60	55	-793.19	8.50
Sex	Time	1	Time	-432.24	37	-790.49	11.20
Sex	Time	1	1	-405.94	19	-773.89	27.80

Table S2: Model selection table for standard Jolly-Seber model, indicating formulas for each parameter: population size *N*, entry probability β, survival probability ϕ and capture probability *p*. Log likelihood *LogL*, number of parameters *k*, Akaike's Information Criterion *AIC*, and difference between top ranked model (by AIC) and all other models *ΔAIC*, is given for each model. The top 10 best fitting models (by AIC) are given as well as the models where either ϕ or *p* are constant or time dependent (where these were not within the 10 best models).

N	$\boldsymbol{\beta}$	$\boldsymbol{\phi}$	***p***	***LogL***	***k***	***AICc***	***ΔAICc***
Sex	Time	Time	Time	-602.82	60	-1085.64	0
Sex	Time	1	Time	-583.48	42	-1082.96	2.68
Sex	Time	Sex	Time	-583.62	43	-1081.23	4.41
Sex	Time	Time	1	-565.31	41	-1048.63	37.01
Sex	Time	Time	Moon	-566.24	42	-1048.48	37.16
Sex	Time	Time	Sex	-565.66	42	-1047.32	38.32
Sex	Time	Time	Effort	-565.46	42	-1046.92	38.72
Sex	Time	Time	Moon + Effort	-566.17	43	-1046.33	39.31
Sex	Time	Time	Sex + Moon	-566.76	44	-1045.52	40.12
Sex	Time	Time	Temp	-564.71	42	-1045.43	40.21
Sex	Time	1	1	-493.13	23	-940.26	145.38

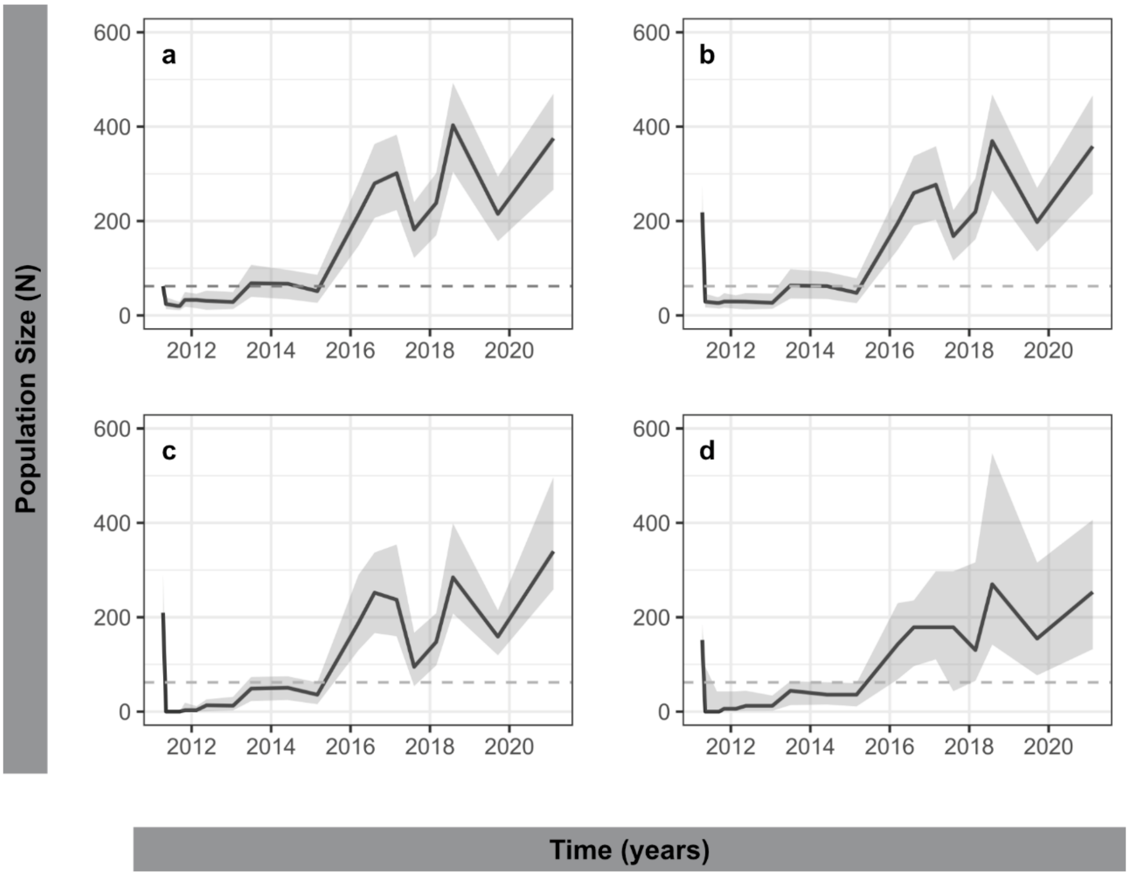

Figure S1: Comparison of abundance estimates from four formulations of the Jolly-Seber model: a) our translocation model which accounts for known initial population size; b) the standard POPAN formulation of the Jolly-Seber model; c) the *POPAN* model provided in the R package *RMark* (Laake 2013); d) the *JS* model provided in the R package *marked* (Laake *et al.* 2013). Abundance estimates are for the population of lesser night gecko (*Nactus coindemirensis)* on Ile Marianne, between the translocation of 62 adults in April 2011 (indicated by the grey dashed line) and June 2021. The model had time dependent β and ϕ and constant p.

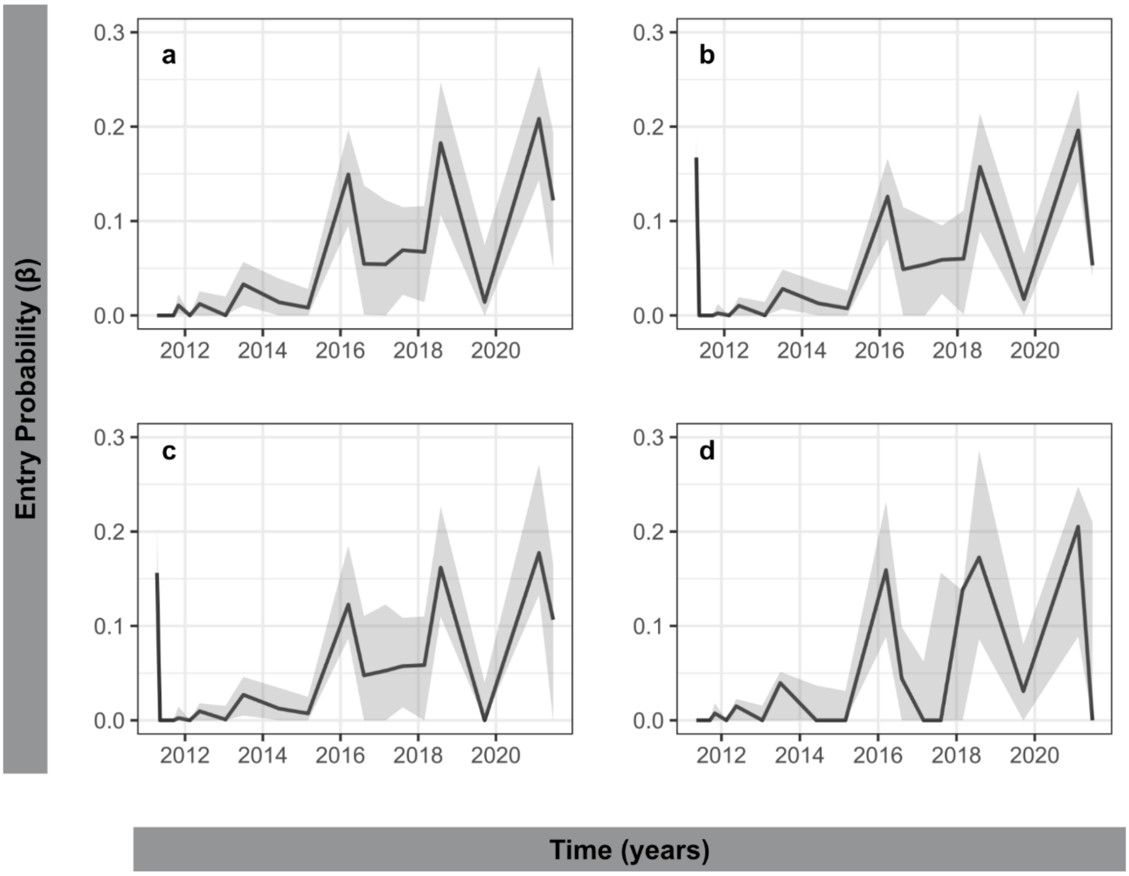

Figure S2: Comparison of entry probability β estimates from four formulations of the Jolly-Seber model: a) our translocation model which accounts for known initial population size; b) the standard POPAN formulation of the Jolly-Seber model; c) the *POPAN* model provided in the R package *RMark* (Laake 2013); d) the *JS* model provided in the R package *marked* (Laake *et al.* 2013). Estimates are for the population of lesser night gecko (*Nactus coindemirensis)* on Ile Marianne, between the translocation of 62 adults in April 2011 and June 2021. The model had time dependent β and ϕ and constant *p*.

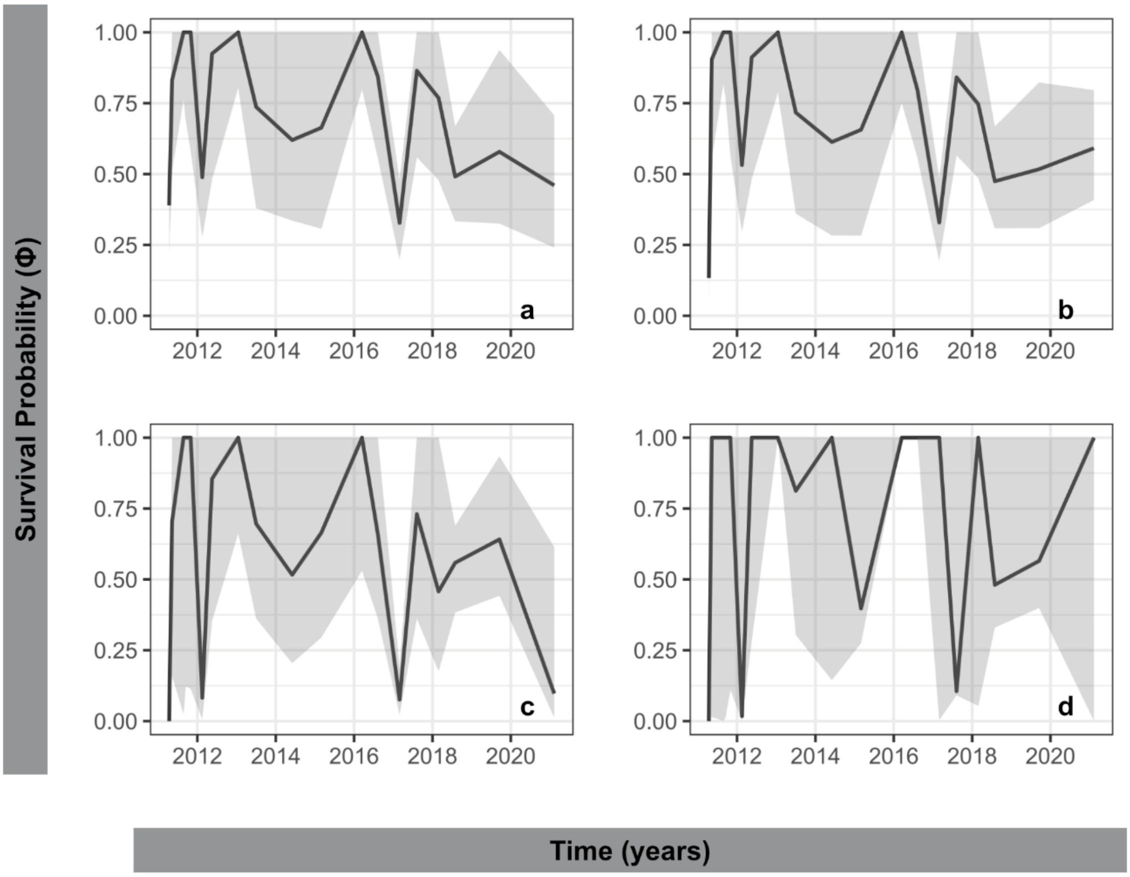

Figure S3: Comparison of survival probability ϕ estimates from four formulations of the Jolly-Seber model: a) our translocation model which accounts for known initial population size; b) the standard POPAN formulation of the Jolly-Seber model; c) the *POPAN* model provided in the R package *RMark* (Laake 2013); d) the *JS* model provided in the R package *marked* (Laake *et al.* 2013). Estimates are for the population of lesser night gecko (*Nactus coindemirensis)* on Ile Marianne, between the translocation of 62 adults in April 2011 and June 2021. The model had time dependent β and ϕ and constant *p*.

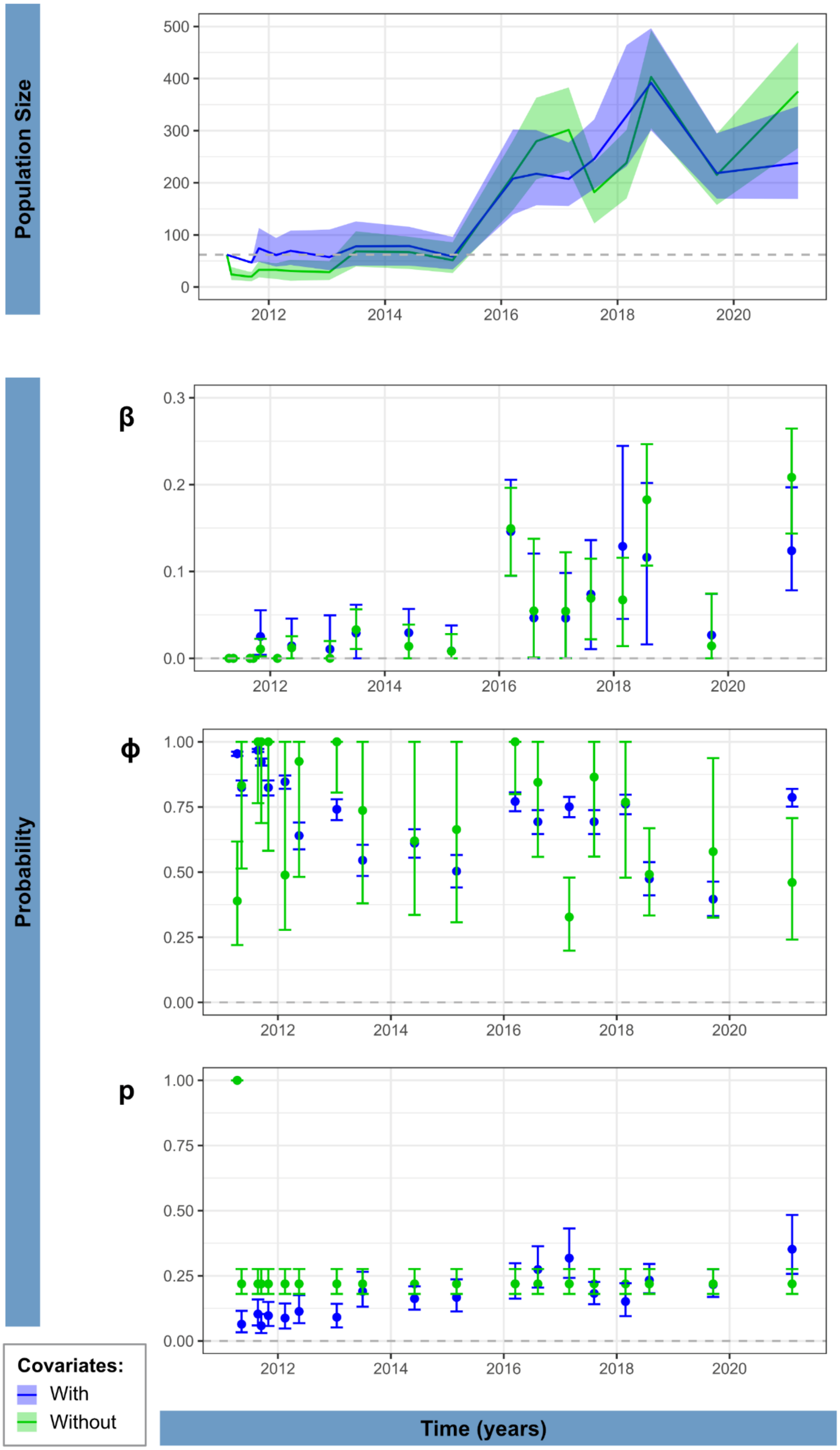

Population Size
500
400
300
200
100
0
2012
2014
2016
2018
2020
Probability
β
0.3
0.2
0.1
0.0
ϕ
1.00
0.75
0.50
0.25
0.00
p
Covariates:
With
Without
Time (years)

Figure S4: Comparison of the estimated parameters of the lesser night gecko population, *Nactus coindemirensis*, on Ile Marianne from the translocation JS model, using the models with lowest AIC with (blue) and without (green) covariates. The model with covariates had time dependent β, constant ϕ and p dependent on survey effort, moon phase and air temperature. The model without covariates had time dependent β and ϕ and constant p.

References

Laake, J.L. 2013. *RMark: An R Interface for Analysis of Capture-Recapture Data with MARK*. AFSC Processed Rep, **1:**25. Alaska Fisheries Science Center, NOAA, Seattle, USA.

Laake, J.L., Johnson, D.S. & Conn, P.B. 2013. "marked: An R package for maximum-likelihood and MCMC analysis of capture-recapture data." *Methods in Ecology and Evolution* 4: 885-890.
